# The way to uncovering and utilizing marine microbial resources

**DOI:** 10.1016/j.engmic.2024.100175

**Published:** 2024-09-24

**Authors:** Zhi-Feng Zhang, Meng Li

**Affiliations:** Archaeal Biology Center, Synthetic Biology Research Center, Shenzhen Key Laboratory of Marine Microbiome Engineering, Key Laboratory of Marine Microbiome Engineering of Guangdong Higher Education Institutes, Institute for Advanced Study, Shenzhen University, Shenzhen 518060, PR China

**Keywords:** Metagenome, MAG, Global ocean microbiome genome catalogue, CRISPR-Cas9, Antimicrobial peptide, PETases

## Abstract

Recently, Chen et al. published their breakthrough results on a marine microbial genomic catalog and genetic potentials in bioprospecting in Nature, providing unprecedented opportunities for development and utilization of genetic resources of marine microorganisms. To highlight this article, we summarized and highlighted their breakthroughs seriatim

## Graphical abstract

**Figure:**
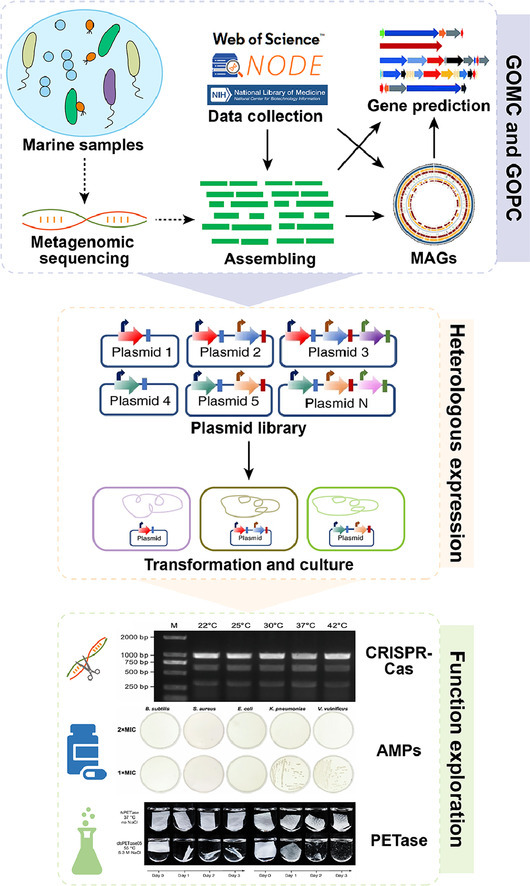

Oceans cover over 70 % of the Earth's surface, and constitute the largest microbial bank on Earth. It is estimated that there are as many as 10^10^ microbial species and 10^29^ microbial cells in the ocean [1,2]. Microorganisms survive throughout the entire ocean, including water, sediment, cold seep, hydrothermal vent, and even the trench and sub-seafloor biosphere. Due to the adaption to various habitats, they are characterized by high taxonomic and metabolic diversity, and are known as an important repository of genetic resources for industry, agriculture, and biomedicine [3,4].

With the rapid development of high-throughput sequencing and molecular biology, omics technology combined with synthetic biology has enabled us to explore and utilize the microbial dark matter without culturing from complex environments [5]. Indeed, recent surveys have greatly expanded the microbial phylogenetic diversity, and revealed that the functional diversity had not been captured previously, especially in the ocean, the largest ecosystem on Earth [3,4,[6], [7], [8]]. The potential of marine microbes as a reservoir of new enzymology and natural products for bioprospecting remains largely underestimated. Recently, Chen et al. [9] published their breakthrough results on a marine microbial genomic catalog and genetic potentials in bioprospecting in *Nature*, providing unprecedented opportunities for development and utilization of genetic resources of marine microorganisms.

Firstly, Chen and colleagues collected and reanalyzed *ca.* 237 Tb metagenomic data of marine microbiomes from diverse marine ecosystems, ranging from the Antarctic to the Arctic, from the coastal to the deep sea, and from the surface ocean to the ultra-deep abyss of 10,000 m, yielding over 43,100 metagenome-assembled genomes (MAGs). Among them, >20,000 microorganisms are potentially newly discovered species, and nearly 10,000 microorganisms are first discovered in unique habitats such as the deep sea. Based on the generated genomic dataset as well as three additional databases, the authors constructed an integrated global ocean microbiome genome catalogue (GOMC), comprising 24,195 non-redundant genomes in species-level. GOMC is three times larger than the recently reported OMD [4] or OceanDNA [10] database of marine genomes. Furthermore, they constructed a global ocean microbiome protein catalogue (GOPC) by predicting open reading frames of the assembled contigs, yielding 2.458 billion unique genes. GOPC is 60 times larger than the protein sequence database of Tara Ocean [3], providing a more comprehensive resource for bioprospecting. By using well-established deep learning algorithms and molecular approaches in the laboratory, the authors then tested the activity, effectiveness and application potentials of candidate genes in GOMC.

1. Genome-editing tools: *cas9* is the most widely used and well-known genome-editing tool [11]. In the study, the team identified 36 new CRISPR-Cas9 genes, and tested the editing capability of Om1Cas9, one *cas9* gene identified from the bacterial genome of *Staphylococcus* found in the Mediterranean Sea. They introduced the gene into a model bacterium and measured an editing efficiency of 94 % *in vitro*.
2. Antimicrobial peptides: The authors identified 117 candidate antimicrobial peptides using a deep-learning approach, and successfully synthesized 63 of them by solid-phase peptide synthesis. Then, the authors examined the antimicrobial activities against five bacterial strains, including some human pathogens, such as *Staphylococcus aureus, Klebsiella pneumoniae* and *Vibrio vulnificus*. Ten of the synthesized peptides exhibited antimicrobial activity against at least one of the five tested strains. Among them, cAMP_87, derived from a novel *Salinibacteraceae* genome, could inhibit the growth of all five microbial strains tested by damaging the bacterial membrane at low peptide concentrations.
3. PETases: Polyethylene terephthalate (PET) is an artificial polymer material that has become a common source of environmental pollution, due to the difficulty in degrading [12]. Here, the authors identified about 1600 homogenous genes similar to bacterial enzyme *Is*PETase encoding genes. Heterologous expression and *in vitro* biochemical characterization determined three halophilic PET hydrolases with superior catalytic activity against amorphous GfPET films from hadal trench and hydrothermal vents water. These three superior PETases displayed 11.8–44.3 times higher catalytic activity than *Is*PETase under optimal incubation condition. Especially, dsPETase05, the PETase derived from hydrothermal vents, achieved an 83 % depolymerization rate in three days, which is much higher than that of *Is*PETase.

Overall, Chen et al. [9] established a feasible way from the construction of a marine microbial genomic catalogue, identification of functional genes, to the detection and application evaluation of gene products in the laboratory (Fig. 1). By large-scale genomic data mining and three independent biotechnological explorations, the work provides us strong evidence for the potential of marine microorganisms and offers a valuable guide for exploring biotechnological and biomedical resources from the vast amount of genomic data.

**Fig. 1:**
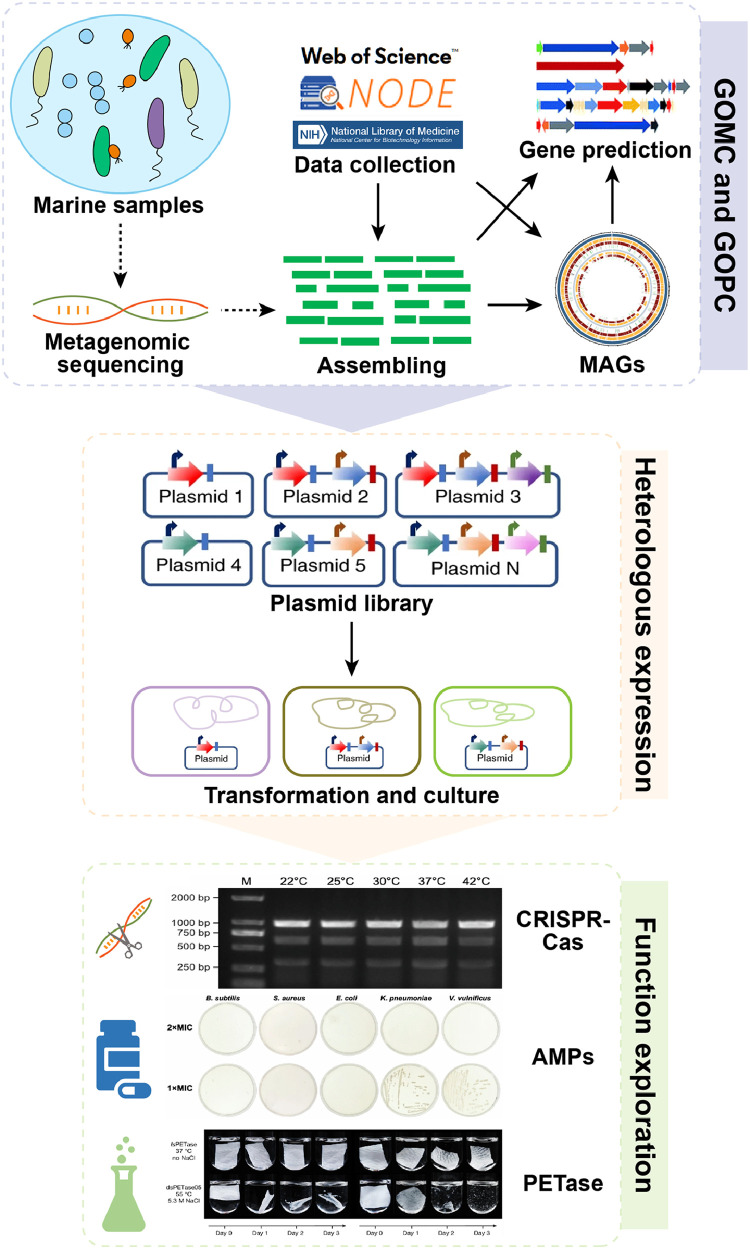
The pipeline from microbial genomes to their function potentials. The dashed arrows in first section indicate steps that were not applied by Chen and his colleagues. GOMC: Global ocean microbiome genome catalogue; GOPC: Global ocean microbiome protein catalogue; MAGs: Metagenome-assembled genomes; AMPs: Antimicrobial peptides.

Although the number of microbial genomes from the planet's ecosystems are increasing rapidly in recent years [13], “how may microorganisms are there” is still one of the most fascinating questions. At this juncture, by systematic genome collection of marine microbiome, Chen and colleagues made a quantum leap toward parsing the diversity of earth microorganisms. This research marks a new milestone in the field of marine metagenomics, highlighting the crucial role of marine microbiomes in improving human well-being and promoting environmental sustainability. These findings not only open a new window for the sustainable exploration and utilization of ocean microbial resources, but also lay the foundation for future biotechnology and biomedical studies.

## CRediT authorship contribution statement

**Zhi-Feng Zhang:** Writing – original draft. **Meng Li:** Writing – review & editing, Supervision, Funding acquisition.

## Declaration of Competing Interest

The authors declare that they have no known competing financial interests or personal relationships that could have appeared to influence the work reported in this paper.
